# Assessing robustness to worst case publication bias using a simple subset meta-analysis

**DOI:** 10.1136/bmj-2023-076851

**Published:** 2024-03-15

**Authors:** Maya B Mathur

**Affiliations:** 1 Quantitative Sciences Unit and Department of Pediatrics, Stanford University, Palo Alto, CA 94304, USA

## Abstract

This article discusses a simple method, known as a meta-analysis of non-affirmative studies, to assess how robust a meta-analysis is to publication bias that favors affirmative studies (studies with significant P values and point estimates in the desired direction) over non-affirmative studies (studies with non-significant P values or point estimates in the undesired direction). This method is a standard meta-analysis that includes only non-affirmative studies. The resulting meta-analytical estimate corrects for worst case publication bias, a hypothetical scenario in which affirmative studies are almost infinitely more likely to be published than non-affirmative studies. If this estimate remains in the same direction as the uncorrected estimate and is of clinically meaningful size, this suggests that the meta-analysis conclusions would not be overturned by any amount of publication bias favoring affirmative studies. Meta-analysis of non-affirmative studies complements an uncorrected meta-analysis and other publication bias analyses by accommodating small meta-analyses, non-normal effects, heterogeneous effects across studies, and additional forms of selective reporting (in particular, P-hacking).

Meta-analytical evidence contributes substantially to clinical guidelines and policy, but the results of meta-analyses can be severely compromised by publication bias. Such bias can occur, for example, if studies that support a given hypothesis are more likely to be published than studies with null or negative results.^1^ Assessing the robustness of results to potential publication bias is therefore a key component of performing a meta-analysis.^1^ However, a review of recent meta-analyses in high impact medical journals found that 55% did not assess publication bias at all.^2^ Of meta-analyses that did assess publication bias, most (85%) exclusively considered whether there was asymmetry in funnel plots, which plot the point estimates versus standard errors from studies (fig 1).^2^ Asymmetry in funnel plots is often assessed visually, or by using statistical methods such as Egger’s regression,^4^ trim and fill,^5^ precision effect test (PET),^6^ or precision effect test and precision effect estimate with standard errors (PET-PEESE).^6^ These methods assess whether there are small-study effects, which occur when small studies tend to have larger point estimates than large studies.^4^ ^5^ As the methods’ originators and others have noted, small-study effects might reflect not only publication bias but also genuine scientific differences between smaller and larger studies.^2^ ^4^ ^7^ ^8^ For example, the most effective interventions might be used in smaller studies if such interventions are more expensive to implement.^2^

**Fig 1:**
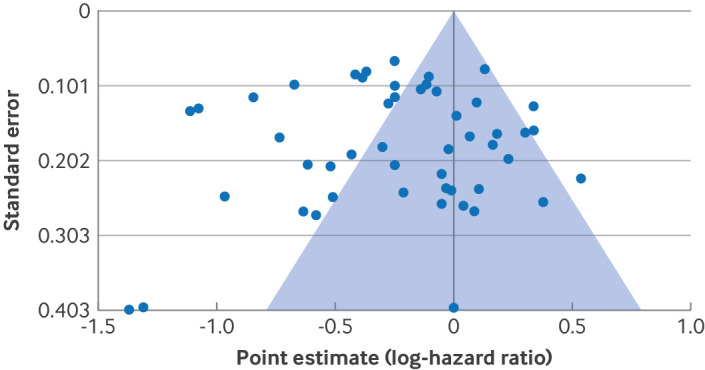
Basic funnel plot for a meta-analysis by Li et al^3^

Despite longstanding directives about interpreting funnel plots and related methods, medical researchers do routinely use them with the intention of assessing publication bias.^2^ ^9^ Interpreting funnel plot asymmetry in this manner requires making implicit assumptions about how effect sizes are distributed across studies and about the mechanism of publication bias. In papers that use funnel plots methods, these assumptions are rarely made explicit. Furthermore, in many meta-analyses, the assumptions might not capture the way publication bias actually operates. The key assumptions are that the publication process favors large point estimates rather than significant P values, and that it does not affect the largest studies. Also, when interpreted as tests of publication bias, some methods based on funnel plots can perform inadequately for small meta-analyses or when effects differ across studies (heterogeneity).^2^ ^7^ ^8^ Last, these methods can perform poorly if the estimates included in the meta-analysis are statistically dependent, which can occur if, for example, the meta-analyst includes multiple estimates from the same study.^10^ I concur with others that funnel plot methods can be useful to investigate small-study effects in general, but that the methods should not be interpreted as specific assessments of publication bias.^7^

Other methods are specifically designed to investigate or to correct for publication bias. For example, selection models involve assuming a certain model of how publication bias operates and then fitting a model that estimates the usual meta-analytical quantities of interest (the mean and heterogeneity of population effect sizes) as well as the strength of the publication bias itself. Numerous selection models exist, but perhaps the most widely used is the two-step selection model.^11^ This model assumes that studies with significant P values and positive estimates are more likely to be published than studies with non-significant P values or negative estimates. If the publication process is thought to favor negative-signed estimates rather than positive-signed estimates, then the same definitions apply but with “positive” and “negative” reversed. 

Other selection models make different assumptions about the mechanism of publication bias^12^ ^13^; some, for example, assume that a study’s probability of being published is a continuous function of the study’s one-tailed P value.^13^ Selection models are statistically well justified and accommodate effect heterogeneity, and we recommend that their results be reported more routinely in meta-analyses.^2^ However, these methods have their own limitations and assumptions. In particular, most selection models assume that the true effect sizes (before any publication bias occurs) are normally distributed and independent, and the models can perform poorly when these assumptions are violated.^10^ Also, these methods can require a large number of studies to perform well—this is especially the case for selection models that accommodate more complex forms of publication bias.^14^ ^15^

Other methods for publication bias are essentially hybrids of methods based on the funnel plot and selection models.^16^ ^17^ For example, robust bayesian model-averaging (RoBMA) involves specifying prior beliefs in the plausibility of various publication bias models (including selection models and the models underlying PET and PET-PEESE) and essentially averaging over the results in a manner that accounts for model fit given the data.^16^ These hybrid methods can provide some additional flexibility compared with funnel plot methods or selection models alone, but they also share many of their limitations. For example, RoBMA can perform worse than an uncorrected meta-analysis in the presence of P-hacking in addition to publication bias.^18^ The relative performance of funnel plot methods, selection models, and hybrid methods is further discussed below.

All of these standard methods specifically assess publication bias that acts as a filter for which studies are published and ultimately included in the meta-analysis. However, selective reporting can also occur within studies. Researchers might P-hack by fitting different models to the same dataset or by analyzing several outcomes in an attempt to obtain an affirmative estimate.^19^ ^20^ ^21^ In fact, such P-hacking is quite common, even according to researchers’ self-admissions.^21^ When there is both traditional publication bias and P-hacking, standard methods for publication bias can be severely biased in either direction.^18^

This article describes a simple and complementary method known as a meta-analysis of non-affirmative studies (MAN). MAN is an additional method to assess how robust a meta-analysis might be to publication bias as well as many forms of P-hacking. This method is not intended to replace existing ones but rather to serve as a useful addition that can provide complementary insights while allowing for small meta-analyses, non-normal effects, and dependent effect sizes. This article describes why conducting a meta-analysis of non-affirmative results is a conservative sensitivity analysis; that is, it characterizes robustness to a hypothetical, worst case form of publication bias. The article then describes how to conduct and interpret MAN in practice, illustrated by reanalysis of three previously published meta-analyses.

**Summary points**

Meta-analytical evidence contributes substantially to clinical guidelines and policy, but the results of meta-analyses can be severely compromised by publication bias
Meta-analysis of non-affirmative studies is a simple method to conservatively assess the robustness of meta-analysis results to publication bias
To perform this method, a standard meta-analysis is conducted of only studies that are non-significant or have estimates in the undesired direction
As a conservative sensitivity analysis, this approach complements existing methods by accommodating additional forms of selective reporting, small meta-analyses, and heterogeneous effects that might not be normal

## Meta-analysis of non-affirmative results

Conducting MAN involves simply meta-analyzing only the non-affirmative studies and excluding all affirmative studies.^18^ ^22^ No special statistical methods are required—a standard meta-analysis is simply conducted of only the non-affirmative studies. This method is a sensitivity analysis rather than a method for bias correction—that is, the method does not estimate the actual strength of publication bias, but instead assesses how results might change for a certain amount of publication bias. Specifically, MAN provides a meta-analytical estimate that corrects for a worst case form of publication bias in which affirmative studies (studies with significant P values and estimates in the desired direction) are more likely to be published than non-affirmative studies (studies with non-significant P values or estimates in the undesired direction). Instead of estimating how strong publication bias actually is in the meta-analysis, the meta-analytical estimate from MAN corrects for a hypothetical worst case situation in which publication bias favors affirmative studies almost infinitely more than non-affirmative studies.^22^

To understand how this works, assume that affirmative studies were known to be three times as likely to be published as non-affirmative studies. To counteract this threefold favoring of affirmative studies in the publication process, a bias-corrected meta-analysis would need to weight the published non-affirmative studies three times as much as the affirmative studies.^22^ This weighting method is essentially the same as methods routinely used to correct survey samples for non-representative sampling. In practice, however, it is not known how much publication bias has occurred, so instead, the worst case publication bias can be conservatively considered. Following the same logic, if affirmative studies were infinitely more likely to be published than non-affirmative studies, a bias-corrected meta-analysis would need to weight the published non-affirmative studies infinitely more than the affirmative studies—which corresponds to analyzing only the non-affirmative studies.^22^

## Advantages of conducting a meta-analysis of non-affirmative studies 

Reporting the worst case estimate from MAN, along with existing publication bias methods, has several advantages. First, the worst case estimate allows for a conservative consideration of how results might change in the presence of worst case publication bias. In practice, publication bias will rarely be this severe, but if the worst case estimate remains in the same direction as the uncorrected estimate, and remains of clinically meaningful size (ideally also with a reasonably precise confidence interval that excludes the null), then this provides strong evidence that the results would not be overturned by any amount of publication bias that might be present. Although the worst case estimate is highly conservative by design, it is often still informative in practice: for 66% of meta-analyses sampled across scientific disciplines, the worst case estimate agreed in direction with the uncorrected estimate, and for 25% of meta-analyses, its confidence interval also excluded the null.^23^ If the worst case estimate is near the null or is in the opposite direction from the uncorrected estimate, however, this suggests that the meta-analysis might not be robust to worst case publication bias, and then robustness to less extreme publication bias could be assessed by conceptually related sensitivity analyses (section 2, supplement).^22^

As noted previously, MAN can be applied to meta-analyses with characteristics that can compromise the performance of standard methods for publication bias (eg, due to heterogeneity, non-normal effects, small number of studies, or dependent effects).^1^ ^4^ ^8^ ^24^ Additionally, applying MAN can help deal with not only publication bias but also other forms of selective reporting that occur within rather than across studies. In particular, if the studies are P hacked in a manner that favors affirmative results, then the MAN estimate is still conservative (ie, attenuated towards the null). In contrast, standard methods for publication bias could be severely biased either towards or away from the null.^18^ Thus, MAN can provide a more holistic assessment of robustness to multiple forms of selective reporting than do standard methods for publication bias. For example, a large simulation study assessed the performance of MAN in over 80 scenarios exhibiting numerous forms of selective reporting (comprising both publication bias and P-hacking), including many that were more complicated than the simple model assumed by MAN.^18^ These simulations found that the performance of MAN was robust to numerous plausible departures from its assumptions, whereas comparison methods (two-step selection models, PET-PEESE, and RoBMA) often had substantially compromised performance in scenarios with P-hacking.^18^ Section 1 of the supplement describes details of these results.

## How to conduct a meta-analysis of non-affirmative studies

Conducting MAN in practice is straightforward. Meta-analysts first need to decide on substantive grounds which direction of estimates—those greater than the null or those less than the null—are likely to be published preferentially. For example, if the exposure or intervention of interest is thought to be positively associated with the outcome, then publication bias might favor estimates that are greater than the null. In this case, affirmative studies would be defined as those with significant P values and estimates greater than the null, and non-affirmative studies would be those with non-significant P values or estimates less than the null. Alternatively, if the exposure is thought to be negatively associated with the outcome, then publication bias might favor estimates that are less than the null. In this case, affirmative studies would be those with significant P values and estimates less than the null. The next step would be to conduct a standard meta-analysis that includes only the non-affirmative studies. In practice, MAN can be conducted using any standard method for random effects or fixed effects meta-analysis.^25^ ^26^ ^27^ We would generally suggest using robust methods for random effects meta-analysis that do not require population effect sizes to be normal, accommodate dependent effect sizes, and perform well for small meta-analyses.^28^ ^29^ ^30^ These methods, known as robust variance estimation, are easy to implement in R using the packages robumeta^31^ or clubSandwich.^32^

**Fig 2:**
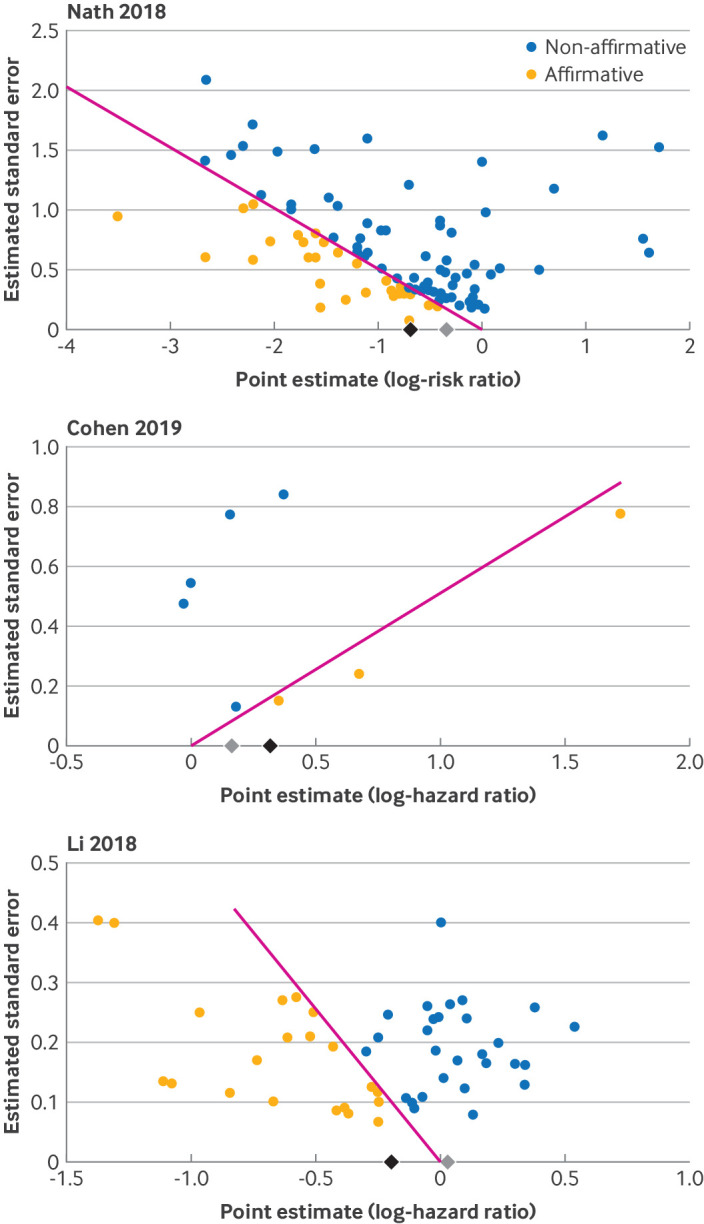
Significance funnel plots for three example meta-analyses.^3^ ^33^ ^34^Studies on the pink diagonal lines have P values exactly at the threshold α=0.05. Black diamond on x axis=uncorrected estimate in all studies; grey diamond on x axis=worst case estimate from meta-analysis of non-affirmative studies; yellow points=affirmative studies; blue points=non-affirmative studies

Along with the numerical results of the MAN analysis, we suggest that meta-analysts create a modified funnel plot, known as the significance funnel. This plot can be created using the R package PublicationBias^22^ or an online web tool (https://metabias.io/). Like a standard funnel plot, the significance funnel plot shows studies’ point estimates against their standard errors (eg, fig 2). But whereas a standard funnel plot focuses on detecting correlation between studies’ estimates and their standard errors, the significance funnel plot focuses on portraying the extent to which the worst case MAN estimate differs from the uncorrected estimate. Thus, the significance funnel differentiates affirmative studies (yellow points) from non-affirmative studies (blue points). The significance funnel also shows the uncorrected meta-analytical estimate (black diamond on x axis) and the MAN estimate (grey diamond on x axis). As a simple rule of thumb, if the diamonds are close to one another, this suggests that the meta-analysis is relatively robust to worst case publication bias. In contrast, if the diamonds are far apart or if the grey diamond represents an effect size that is too small to be clinically meaningful, the meta-analysis might be sensitive to worst case publication bias. However, the meta-analysis might still be robust to less severe publication bias, which researchers can assess using follow-up sensitivity analyses (section 2, supplement).^22^

Although our focus is not on the design of meta-analyses in general, a few points merit special attention in the context of publication bias and MAN. First, as a general principle for meta-analysis, inclusion criteria for primary analyses should generally be designed to exclude studies of low methodological quality. This practice is not yet commonplace, especially in meta-analyses of non-randomized studies.^35^ ^36^ However, using methodological inclusion criteria can help reduce internal bias in the meta-analysis overall, which can occur independent of publication bias.^36^ In the context of conducting MAN, this practice also helps alleviate the possibility that the non-affirmative studies will be of low quality. Second, to characterize differences between affirmative and non-affirmative studies, meta-analysts could report risk-of-bias ratings (eg, ROBINS-I^37^) and key methodological characteristics,^36^ key study level moderators (eg, patient population), and typical sample sizes. These characteristics could be reported separately for all studies, for affirmative studies, and for non-affirmative studies, which would be a simple extension for existing best practices.^38^ More technical points regarding heterogeneity are described in the supplement (section 3).

## Examples

We illustrate applying and interpreting MAN using three published meta-analyses (fig 2). First, a meta-analysis by Nath et al assessed the effect of using atraumatic needles versus conventional needles for lumbar puncture on multiple outcomes.^33^ Our reanalysis of studies on one of the outcomes—the occurrence of any type of headache—included 99 randomized studies, of which 31 were affirmative (favoring atraumatic needles) and 68 were non-affirmative. The uncorrected estimate was risk ratio 0.50 (95% confidence interval 0.44 to 0.58; P*<*0.001). The worst case MAN estimate was 0.71 (0.64 to 0.79; P*<*0.001), which suggests that both the point estimate and confidence interval are quite robust to even worst case publication bias that favors protective effects of atraumatic needles.

Second, Cohen et al’s meta-analysis assessed the association of white coat hypertension (ie, high blood pressure occurring only when the patient is at the doctor’s office) with cardiovascular events.^34^ Their analysis included eight non-randomized studies, of which three were affirmative (suggesting a detrimental association) and five were non-affirmative. On reanalysis, the uncorrected estimate was hazard ratio 1.37 (95% confidence interval 0.95 to 1.98; P=0.07); the worst case estimate was 1.18 (0.84 to 1.66; P=0.12). The authors correctly reported that funnel plot methods can perform poorly for this relatively small number of studies, so they did not assess publication bias. Nevertheless, MAN can be applied and suggests that even under worst case publication bias, the pooled estimate would remain in the detrimental direction (hazard ratio 1.18), although the wide confidence interval includes the null due to the small number of non-affirmative studies.

Third, Li et al’s meta-analysis^3^ assessed the effect of PI3K/AKT/mTOR pathway inhibitors compared with various control treatments on progression-free survival in patients with advanced solid tumors. Their analysis included 50 estimates that were clustered within 39 randomized studies. Of the estimates, 22 were affirmative (suggesting protective effects) and 28 were non-affirmative. On reanalysis, the uncorrected estimate was hazard ratio 0.82 (95% confidence interval 0.74 to 0.91; P*<*0.001), but the worst case estimate was close to the null (1.03; 0.94 to 1.12; P=0.49). This suggests that the results might not be robust to worst case publication bias that favors protective effects; follow-up sensitivity analyses could then be conducted (section 2, supplement).^22^

## Discussion

Key advantages of MAN are its applicability to meta-analyses that have heterogeneous and even non-normal effects, dependent effects, a small number of studies, and P-hacking in addition to publication bias. Funnel plot methods and other standard methods for publication bias can perform poorly in these situations.^1^ ^4^ ^8^ ^18^ ^24^ MAN also has certain conceptual strengths. MAN assumes that publication bias favors significant studies with estimates in the desired direction and that all studies, regardless of size, can be affected by publication bias. In contrast, funnel plot methods effectively assume that publication bias favors large point estimates, rather than significant P values, and that the largest studies are not affected by publication bias at all.^4^ Because MAN considers a different form of publication bias—one that is supported by empirical findings on how investigators interpret and report P values^39^ ^40^ ^41^—it can provide different insights than do funnel plots. Selection models can also consider this form of publication bias,^11^ and as described in the introduction, we recommend routinely reporting results of such models as well.^2^

MAN also has limitations. The method assumes that publication bias (and P-hacking, if present) favor affirmative results but that, among non-affirmative results, it does not favor larger point estimates.^18^ ^22^ In other words, non-affirmative results with larger point estimates are no more likely to be published than other non-affirmative results with smaller point estimates. The simulation results mentioned above do suggest that the method is quite robust to numerous departures from these assumptions, but simulations cannot be exhaustive. In practice, we suggest examining diagnostic plots, such as the density of studies’ z scores, to help assess whether this assumption is plausible.^18^ ^23^ Additionally, MAN cannot be applied to meta-analyses that contain only affirmative studies, and if there are very few non-affirmative studies, then its confidence interval could be wide. Last, because MAN considers only worst case publication bias, if the worst case estimate is near the null, the meta-analysis might nevertheless be robust to less severe publication bias. Such an analysis is inconclusive, and in this case, we would suggest conducting the sensitivity analyses mentioned above to consider less extreme publication bias and describe the amount of publication bias that would be required to explain away the results (section 2, supplement).^22^ However, again, empirical evidence suggests that for many meta-analyses—although certainly not all—MAN might in fact suggest robustness to even worst case publication bias.^23^

In summary, MAN could be routinely reported in meta-analyses to help assess robustness to worst case publication bias or P-hacking that favors affirmative results, ideally along with a significance funnel plot. MAN complements an uncorrected meta-analysis and standard publication bias analyses by accommodating effects that differ across studies, small meta-analyses, non-normal effects, and additional forms of selective reporting.

## Web extra

Extra material supplied by authors

Web appendix: Supplement

## Notes

Provenance and peer review: Not commissioned; externally peer reviewed.
