# Supplementary material for: Assessing robustness to worst case publication bias using a simple subset meta-analysis
Source: BMJ. 2024 Mar 15;384:e076851. doi: 10.1136/bmj-2023-076851 (PMC10941077; doi:10.1136/bmj-2023-076851)
Supplement: Supplementary file 1 — Web appendix: Supplement [file matm076851.ww.pdf]

Supplement

Contents

1 Summary of simulation studies **2**

2 Related sensitivity analyses and bias-correction methods **3**

3 Technical points regarding heterogeneity **5**

1. Summary of simulation studies

As mentioned in the Introduction, a large simulation study assessed the performance of MAN in over 80 scenarios exhibiting realistic forms of selective reporting (potentially comprising both publication bias and p-hacking). Many of the simulated forms of selective reporting were more complicated than the simple model assumed by MAN [1]. The mechanisms of p-hacking were replicated from a recent simulation framework and R package, `phackR` [2, 3]. This framework simulates p-hacking in ways that its authors argued are realistic and widespread: selecting among dependent variables, selecting among subgroup analyses, and optional stopping [2]. Additionally, the simulation study assessing MAN also included several forms of publication bias and p-hacking, including those in which estimates in both directions were favored (not only positive estimates), and those in which smaller p-values were favored even within the categories of affirmative and nonaffirmative [1]. These simulations also examined performance for meta-analyses with varying numbers of studies (including meta-analyses with as few as 10 nonaffirmative studies), varying amounts of heterogeneity, normal and skewed population effect sizes, and statistically independent or dependent estimates across studies.

The results indicated that the performance of MAN was largely robust to these different forms of publication bias and p-hacking, meaning that the method remained conservative even when its assumptions were violated. It is important to note that although these simulations assessed numerous forms publication bias and p-hacking, these forms were not exhaustive. Of the comparison correction methods, two-step selection models typically outperformed PET-PEESE and RoBMA. Two-step selection models typically performed better than an uncorrected meta-analysis, but was typically more biased than MAN (although MAN is intended to be conservative rather than unbiased) and had wider confidence intervals.

Perhaps surprisingly, PET-PEESE and RoBMA often (though not always) performed worse than an uncorrected meta-analysis. These results in part reflect these methods' assumption that there is publication bias but not p-hacking, whereas MAN accommodates both. PET-PEESE was typically extremely negatively biased and imprecise, which may in part reflect the lack of variation in study standard errors in the `phackr` simulation environment [4]. On the other hand, in other simulations with SWS and greater variation in standard errors, PET-PEESE again showed substantial, though less extreme, negative bias [5]. RoBMA was often substantially biased, though less so than PET-PEESE. We speculate that this issue could reflect RoBMA's prior structure, in which models that are nested within other models in the suite (e.g., selection models with different numbers of steps) are also included as separate models. Two-step selection models were considerably more robust to the additional presence of p-hacking than were PET-PEESE and RoBMA. We give a more detailed explanation for these findings regarding other methods in the accompanying methodological paper [1].

Two other simulation studies explored the performance the more general sensitivity analysis frameworks of which MAN is a special case [6, 7]. One simulation study yielded similar conclusions in 12,480 scenarios that included yet more forms of publication bias and p-hacking,

as well as many other variations on characteristics of the meta-analyses [7]. Another simulation study also yielded similar conclusions, and furthermore found that the generalizations of MAN remained valid in meta-analyses with even fewer studies (e.g., fewer than 5), although the confidence intervals can become quite wide [6].

2. Related sensitivity analyses and bias-correction methods

We now consider several related sensitivity analyses that can performed in addition to MAN. All can be conducted using the online web tools available at `https://metabias.io/`, or alternatively using R packages listed below.

Sensitivity analysis for less severe publication bias

First, as noted above, because MAN is designed to be conservative, if the worst-case estimate is near the null or is in the opposite direction from the uncorrected estimate, this does not indicate that the results are not robust to lesser amounts of publication bias. In such cases, we recommend additionally conducting Mathur & VanderWeele's [6] sensitivity analysis, which characterizes how severe publication bias would need to be in order to explain away the meta-analysis results (i.e., for the true average effect across studies to be null, or alternatively any other chosen value). That is, this sensitivity analysis characterizes how many times higher the publication probability for affirmative studies (vs. nonaffirmative studies) would need to be in order for the true average effect to be null. This ratio of publication probabilities is called "S" for "selection ratio".

For example, recall that in the aforementioned meta-analysis by Li et al. [8], the worst-case estimate was close to the null ($HR = 1.03$; 95% CI: [0.94, 1.12]). Mathur & VanderWeele's [6] sensitivity analysis indicates, more specifically, how much publication bias would be required to explain away the results. In this case, the required amount of publication bias is fairly strong: affirmative studies would need to be $S = 8$ times more likely to be published than nonaffirmative studies [6]. These sensitivity analyses can also characterize the severity of publication bias required to shift the confidence interval for the meta-analytic estimate to include the null. For the meta-analysis by Li et al. [8]. affirmative studies would need to be 2 times more likely to be published in order to shift the confidence interval to include the null [6]. After conducting such sensitivity analyses, the meta-analyst should then consider on substantive grounds whether this much publication bias is plausible in the meta-analysis at hand. Such assessments should reflect the methods used in the meta-analysis, such as the sensitivity of the search strategy and inclusion of grey literature, and could also be informed by existing empirical results regarding typical strengths of publication bias in meta-analyses from different disciplines [9]. Such sensitivity analyses can be conducted using the R package `PublicationBias` [6].

Sensitivity analysis for publication bias and internal bias

Additionally, meta-analyses can be compromised by studies' internal biases (e.g., confounding in nonrandomized studies) as well as publication bias [10]. These biases often operate nonadditively: publication bias that favors affirmative results selects indirectly for studies with stronger, positive internal bias. Sensitivity analyses related to MAN can help address the questions: (1) "For a given severity of internal bias across studies and of publication bias, how much could the results change?"; and (2) "For a given severity of publication bias, how severe would internal bias have to be, hypothetically, to attenuate the results to the null or by a given amount?"

We consider two simple examples of how these methods can be applied. First, suppose we assume that all studies could have internal bias, though potentially of varying degrees. If we consider worst-case publication bias, then the average amount of internal bias required to explain away the result is simply equal to the worst-case estimate itself [7]. This is simply because the studies' internal biases would have to be strong enough to cancel out the worst-case estimate. Second, suppose that a known subset of studies have internal bias from a given source (e.g., the nonrandomized studies may have confounding bias), while the rest do not (e.g., randomized studies). Considering worst-case publication bias, the average amount of internal bias required to explain away the result is equal to the worst-case estimate divided by a measure of how much of the "information" in the nonaffirmative studies (more specifically, statistical precision) is contributed by nonrandomized studies. These sensitivity analyses can be conducted using the R package `multibiasmeta` [7].

Bias correction for publication bias and p-hacking

Like most methods for publication bias, the aforementioned methods for publication bias with or without internal bias [6, 7] do not account for p-hacking. When p-hacking is of particular concern, then in addition to using MAN to obtain a conservative estimate, one can use a related approach, called "right-truncated meta-analysis" (RTMA), to obtain a bias-corrected estimate that accounts for both publication bias and p-hacking that favors affirmative results. Like MAN, RTMA analyzes only the nonaffirmative studies. However, unlike MAN, RTMA is not a simple subset meta-analysis, but rather uses the nonaffirmative studies to impute the entire underlying distribution of population effect sizes, yielding a bias-corrected estimate rather than a conservative estimate [1].

For example, Lodder et al. [11] conducted a meta-analysis to investigate the psychological theory of money priming, which postulates that exposure to money-related stimuli (e.g., images of paper currency) may affect a diverse range of attitudes and behaviors, such as propensity to help others and propensity to cheat. A large published literature on money priming has appeared to support the theory, but the extent to which such findings are artifacts of both publication bias and p-hacking has been contested [12]. Lodder et al. [11] meta-analyzed 287 psychology experiments that manipulated whether participants were

exposed to a money prime versus to a control prime that was not related to money. Studies could measure any psychological or behavioral outcome. The uncorrected estimate on the Hedges' g scale was $g = 0.26$ (95% CI: [0.21, 0.30]), which supports money priming effects, but the worst-case MAN estimate was null ($g = 0$; 95% CI: $[-0.03, 0.02]$) [1]. To supplement this conservative estimate, we additionally used RTMA to obtain an estimate that is bias-corrected for both publication bias and p-hacking. This estimate was also null ($g = 0.03$; 95% CI: $[-0.01, 0.09]$), suggesting that the uncorrected estimate of $g = 0.26$ may be entirely attributable to publication bias and/or p-hacking [1]. These sensitivity analyses can be conducted using the R package `phacking` [1].

3. Technical points regarding heterogeneity

In the context of publication bias, a key goal of meta-analysis is to estimate the average population effect size across all results that would have been included in the meta-analysis had there been no publication bias. Although this goal cannot be fully attained in practice, MAN is conservative in the sense that it provides a lower bound (in expectation) on what this average population effect size might be. Nonaffirmative and affirmative studies may differ based on substantive characteristics, and the worst-case estimate will reflect the characteristics typical of nonaffirmative studies. For example, consider a meta-analysis of randomized trials of an intervention. If the intervention is more effective in some patient populations (e.g., those with less advanced disease) than others (e.g., those with more advanced disease), then the nonaffirmative studies will tend to be those conducted using patients with more advanced disease. With this form of heterogeneity between nonaffirmative and affirmative studies, MAN still retains its usual interpretation as a conservative estimate. That is, the worst-case estimate will still be smaller than the average population effect across all studies that would have been included in the meta-analysis had there been no publication bias. This is because, with this form of heterogeneity, the average population effect size among all studies is larger than than that among the nonaffirmative studies even if there were no publication bias.

On the other hand, nonaffirmative studies could differ from affirmative studies not only in their substantive characteristics, but also in their susceptibility to biases other than publication bias, such as confounding in nonrandomized studies [7]. Such "internal biases" can occur regardless of whether there is publication bias [7]. In some contexts, nonaffirmative studies might more susceptible to internal biases than affirmative studies if nonaffirmative results reflect, for example, a sloppy implementation of the intervention. Additionally, nonaffirmative studies will often be lower powered. While low power does not inherently create bias, it may be associated with other methodological limitations that do create bias. In the next section, we discuss design and reporting considerations to help mitigate and assess this possibility. In other contexts, nonaffirmative studies might typically be less susceptible to internal bias. For example, such studies are more likely to be preregistered [13, 14], a practice that is likely to improve study conduct and quality in general [15]. As another example, if some meta-analyzed studies are nonrandomized studies that are positively biased due to confounding, while others are randomized (and so are less prone to confounding bias), then the nonaffirmative studies

will typically also have less confounding bias than affirmative studies [7].

References

[1] MB Mathur. "P-hacking in meta-analyses: A formalization and new meta-analytic methods." *Res Synth Methods* (in press). Preprint retrieved from `https://osf.io/ezjsx/`.

[2] Angelika M Stefan and Felix D Schönbrodt. "Big little lies: A compendium and simulation of p-hacking strategies." *Royal Society Open Science* 10.2 (2023), p. 220346.

[3] Angelika M. Stefan. *phackR: Simulate p-Hacking.* R package version 0.0.0.9000. 2023.

[4] Tom D Stanley. "Limitations of PET-PEESE and other meta-analysis methods." *Social Psychological and Personality Science* 8.5 (2017), pp. 581–591.

[5] Maximilian Maier, František Bartoš, and Eric-Jan Wagenmakers. "Robust Bayesian meta-analysis: Addressing publication bias with model-averaging." *Psychol Methods* (2022).

[6] Maya B Mathur and Tyler J VanderWeele. "Sensitivity analysis for publication bias in meta-analyses." *J R Stat Soc Ser C Appl Stat* 69.5 (2020), pp. 1091–1119. URL: `https://doi.org/10.1111/rssc.12440`.

[7] Maya B Mathur. "Sensitivity analysis for the interactive effects of internal bias and publication bias in meta-analyses." *Res Synth Methods* (in press). `https://doi.org/10.1002/jrsm.1667`.

[8] Xuan Li et al. "Efficacy of PI3K/AKT/mTOR pathway inhibitors for the treatment of advanced solid cancers: A literature-based meta-analysis of 46 randomised control trials." *PloS One* 13.2 (2018).

[9] Maya B Mathur and Tyler J VanderWeele. "Estimating publication bias in meta-analyses of peer-reviewed studies: A meta-meta-analysis across disciplines and journal tiers." *Res Synth Methods* 12.2 (2021), pp. 176–191. URL: `https://doi.org/10.1002/jrsm.1464`.

[10] Maya B Mathur and Tyler J VanderWeele. "Methods to address confounding and other biases in meta-analyses: review and recommendations." *Annu Rev of Public Health* 43 (2022), pp. 19–35.

[11] Paul Lodder et al. "A comprehensive meta-analysis of money priming." *Journal of Experimental Psychology: General* 148.4 (2019), p. 688.

[12] Miguel A. Vadillo, Tom E. Hardwicke, and David R. Shanks. "Selection bias, vote counting, and money-priming effects: A comment on Rohrer, Pashler, and Harris (2015) and Vohs (2015)." en. *Journal of Experimental Psychology: General* 145.5 (May 2016), pp. 655–663. ISSN: 1939-2222. DOI: `10.1037/xge0000157`. URL: `http://dx.doi.org/10.1037/xge0000157`.

[13] Christopher Allen and David MA Mehler. "Open science challenges, benefits and tips in early career and beyond." *PLOS Biol* 17.5 (2019), e3000246.

[14] Robert M Kaplan and Veronica L Irvin. "Likelihood of null effects of large NHLBI clinical trials has increased over time." *PLOS One* 10.8 (2015), e0132382.

[15] Maya B Mathur and Matthew P Fox. "Toward open and reproducible epidemiology." *American Journal of Epidemiology* 192.4 (2023), pp. 658–664.
